# Examining Foot Shape Variations in Individuals With and Without Diabetes

**DOI:** 10.1002/jfa2.70060

**Published:** 2025-06-29

**Authors:** Sarah L. Hemler, Robert W. Schuster, A.‐V. Behling, Z. Pataky, L. A. Kelly

**Affiliations:** ^1^ Faculty of Medicine University of Geneva Geneva Switzerland; ^2^ Unit of Therapeutic Patient Education WHO Collaborating Centre Geneva University Hospitals Geneva Switzerland; ^3^ School of Human Movement & Nutrition Sciences The University of Queensland Brisbane Australia; ^4^ School of Health Sciences and Social Work Griffith University Southport Australia; ^5^ Faculty Diabetes Centre Faculty of Medicine University of Geneva Geneva Switzerland

**Keywords:** diabetic foot, peripheral neuropathy, shoe fit, statistical shape modeling

## Abstract

For people with diabetes, a good‐fitting shoe is essential for reducing the risk of ulcers and eventual amputation. However, there is a lack of 3D data explaining differences in foot shape between people with and without diabetes and how individual factors might influence these differences; these data are vital for adequate shoe design and prevention of diabetic foot ulcers. This study quantifies the differences in external foot shapes of people with and without diabetes and peripheral neuropathy and examines which factors might affect these variations. One‐hundred thirty‐six foot scans of older adults with and without diabetes and peripheral neuropathy were used to create and assess foot shape models against demographic and health factors. Principal component analysis (PCA) showed that the feet of people with diabetes and with neuropathy are not necessarily clustered into a particular foot shape but have more pronounced features in specific foot variations (e.g., ankle width, arch height, hallux abduction, and edema vs. atrophic feet) compared to people without diabetes and neuropathy. The mean pairwise distance (intrascore spread) in PC1 and PC2 for individuals with diabetes and neuropathy was 43% larger than for those without diabetes and neuropathy and 24% larger than for those with neuropathy but no diabetes. Partial least squares regression (PLSR) showed potential predicting the presence of diabetes and neuropathy; however, additional data are required to support the trend. Analyses, such as PCA and PLSR, could be useful for determining how to quantify these changes to design more appropriate footwear for these populations.

## 1 Introduction

Diabetes mellitus (DM) affects nearly one in 10 people globally and the prevalence is increasing [1]. It is estimated that 19%–34% of people with diabetes will develop an ulcer at some point in their lifetime [2, 3, 4]. If left untreated, these ulcers often lead to an amputation, accounting for about 85% of amputations in people with diabetes [5]. One of the primary contributing factors to ulcer formation is external stress on the foot, such as high normal and frictional forces, that can be exacerbated by ill‐fitting footwear. Inappropriate footwear has been identified as a principal risk factor for 21%–76% of foot ulcers and/or amputations in the lower extremities for this population [6, 7, 8].

Attention to footwear fit is important for reducing foot deformity [9] and it is especially important for people with diabetes, as foot structure and geometry change with the progression of diabetes and peripheral neuropathy. Roughly half of people with DM have peripheral sensorimotor neuropathy—a lack of pain sensitivity [10]. Intrinsic muscle atrophy, an effect of this neuropathy, is demonstrated by the shortening of the tendons leading to foot deformities such as hammer‐, mallet‐, and claw‐toe formations. These changes along with limited joint mobility lead to higher loading time and force integrals under the heel and the metatarsals [11]. Other structural foot deformities, including pes cavus, pes equinus, and hallux valgus, common in people with diabetes [12], may impact health as shown by the association of hallux valgus angle with ulcer area and infection risk and its link to age and body mass index (BMI) [13]. Foot morphology changes are generally known qualitatively, but there is a gap in the literature quantifying these differences with three‐dimensional (3D) models. Furthermore, there is a lack of understanding of how factors, such as personal health characteristics (e.g., height, weight, and years since diabetes diagnosis) might influence foot shape.

The internal hollow shape of footwear is crafted based on a predefined shoe last, which is assumed to reflect an average foot shape of the wider population. Since all parts of the shoe are designed around this last, ensuring that it accurately represents the population's foot shape is fundamental to creating functional footwear [14]. Currently, lasts that are designed specifically for people with diabetic foot disease are not commercially available. Despite the consideration that foot deformity and poor‐fitting footwear are risk factors for foot ulceration, there is a lack of quantitative detail on how 3D foot shape differs in people with diabetes. This knowledge would enable development of appropriate lasts (footwear) to optimize fit, without expensive individually customized footwear. Previous research has collected unidimensional measurements on the foot (e.g., length and width) to guide shoe last creation [15] and has assessed current shoe fit for people with diabetes [16]. However, as diabetic foot anatomical changes are more complex than simply altering the foot length and width, there is a need for diabetic foot 3D shape characterization and an exploration of how foot shape varies within the clinical population.

Statistical shape models (SSMs) have been widely applied to quantitatively evaluate shape variance in biological systems. SSMs of the external foot shape initially provided a more comprehensive understanding of how healthy feet vary in shape [17] which led to developing a system for detecting and classifying the precise 3D shape abnormalities associated with common foot deformities such as high and low arches and hallux valgus [18]. Attempts have also been made to reconcile dominant features of foot shape variation with foot deformations under load, plantar soft tissue stiffness [19], and foot joint motion and loading during walking and running [20]. Although these studies were able to confirm the previously proposed dominance of prominent shape variations within the healthy population, they also showed that unidimensional metrics are insufficient to capture the complexity of foot shape and its variations.

Two previously defined methods for quantifying shape models are principal component analysis (PCA) and partial least squares regression (PLSR). Studies have used PCA to accurately quantify variations in foot shape and relate foot form to deformation and function [17, 20]. However, PCA has limitations concerning the inclusion of personal health and demographic features in the SSMs. Therefore, PLSR is helpful for understanding the effects of these personal characteristics in the models and has been used in analyses for studying the shape changes of body segments such as the spine [21] and torso [22]. PLSR could be useful for integrating personal characteristics into foot shape exploration.

The aim of this research is to quantify the variance in foot shape within a population of older adults with and without DM and peripheral neuropathy. The goal is to determine how foot shape varies between people with DM and healthy older adults and if specific shape features could predict the presence of DM and peripheral neuropathy. The secondary aim of the research is to explore the ability of certain personal characteristics to identify foot shape. This study is an extension of a previous pilot analysis [23].

## 2 Methods

### 2.1 Participants and Data Collection

A cohort of 69 older adults (described below) from the University of Queensland Healthy Living Center and a private podiatry clinic, both in south‐east Queensland, Australia, was recruited. Participants completed a short questionnaire on demographics (i.e., sex, age, height, and weight), diabetic and foot health for those with diabetes (i.e., time since diagnosis, presence of neuropathy, foot ulcers or surgery, and time actively moving), and footwear fit (i.e., difficulty finding shoes that are comfortable or that fit their feet). A 3D scan of each participant's left and right foot was collected using the FootIn3D scanner (Elinvision, Lithuania; reported accuracy of 0.3 mm) with a 1.4 mm mesh resolution. Participants distributed their weight equally between the scanner and a scale on a raised surface and could support themselves against the adjacent wall. The study protocol was approved by the institutional human research ethics committee of the University of Queensland (2022/HE002010).

### 2.2 Data Processing

**FIGURE 1:**
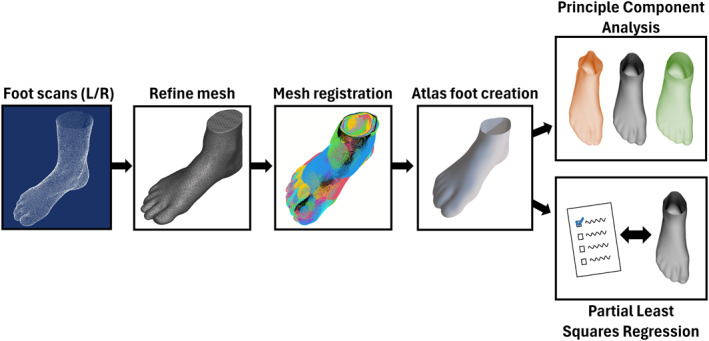
Method for processing the foot scan mesh to perform the two types of analyses.

The scanner produced a 3D point cloud of each foot (Figure 1) from which a triangulated 3D mesh was produced and smoothed using Meshmixer (Autodesk, San Francisco, USA). The mesh was cut above the malleolus, and all left feet were mirrored to create a larger set for exploratory analysis.

Standardizing the meshes such that they contained the same number of identically ordered vertices was performed through a 3D elastic matching algorithm in R (Foundation for Statistical Computing, Vienna, Austria) [24]. The mesh with the surface area closest to the mean surface area (atlas foot) was determined and iteratively morphed such that the mesh fit the shape of all the other meshes. Thus, a set of differently shaped meshes with corresponding 3D vertices was created. General Procrustes Analysis (GPA) was used to remove any remaining differences in scaling along all three dimensions and alignment between the cut, 3D triangulated, morphed meshes, and the atlas mesh.

### 2.3 Principal Component Analysis

Once registered, the dimensionality of the ∼85,000 vertex coordinates (3 dimensions x vertices per foot) was reduced using PCA and the resulting SSMs described the major modes of shape variation, or principal components (PCs), as a set of transformations from each vertex to the corresponding mean vertex. Three SSMs were developed: one containing foot shapes from the two populations (with and without diabetes‐27,689 vertices per foot), one with only the non‐diabetes (ND‐29,244 vertices) population and the other from the diabetic (DM‐27,945 vertices) cohort only. The cumulative eigenvalues of these SSMs were used to assess the percentage of overall shape variability accounted for by each PC [24]. To interpret the PCs in each SSM, the first three PCs were perturbed by ± 3 standard deviations of the corresponding distribution of PC scores.

### 2.4 Partial Least Squares Regression

Partial least squares regression (PLSR) was also performed on the dataset. Response variables were included in the analysis based on the questionnaire data and included three categorical factors (sex, presence of diabetes, and presence of neuropathy) and three continuous factors (age, BMI, and years since diagnosis). The PLSR was performed in MATLAB (Version 2022b, The Mathworks Inc., Natick, MA, USA) [21] and identified latent variables that represented shape features corresponding to largest variations in the response variables. For each analysis, the components that accounted for at least 90% of the variability in the response variables were used to create the model [21].

To control for the innate risk of overfitting in PLSR according to intraperson similarities [25], a leave‐one‐out (LOO) cross validation method was used [21]; all but one pair of feet were incorporated into the model, with the resultant regressions used to predict the characteristics of the omitted foot (either right or mirrored‐left foot). This process was repeated 20 times while randomly assigning the foot being predicted. The results of the randomly omitted foot were then compared to the correctly assigned foot. For binary response variables (e.g., presence of diabetes, sex, or presence of neuropathy), the predicted values (continuous) were converted to a binary value such that values < 0.5 were converted to 0 (absence of the variable) and the remaining were equal to 1 (presence of the variable). The accuracy, sensitivity, specificity, F1 score, area under the receiver operating characteristic curve (AUC), and bookmaker informedness (assessing the classification compared to random guessing) were calculated for the binary variables [21]. AUC was calculated while increasing the binary cutoff from 0 to 1 in 0.05 increments.

## 3 Results

### 3.1 Population

Twenty two people with diabetes (type 2) and 47 people without diabetes participated (Table 1). A two‐sample proportion test showed that there were significantly more women in the nondiabetic population compared to men (*p* = 0.023); other proportions were tested but were not statistically different (Table 1). There was no statistically significant difference in finding comfortable or good‐fitting shoes between the DM and ND populations. None of the participants had experienced prior foot ulcers. In general, the ND group had a lower BMI than the DM group and half of the DM population reported having peripheral neuropathy. A total of 138 feet were scanned; one diabetic foot was excluded from the analysis due to reported anatomy‐altering surgery and one due to a data reconstruction error. Therefore, a total of 136 feet were included in the analyses including all feet. Two subjects (four feet) who did not report their age were excluded from the age‐required analyses.

**TABLE 1:** Descriptive statistics for the populations (DM = people with diabetes and ND = nondiabetic population; % of *n* indicates the % of people within the group for a particular characteristic; ± indicates the standard deviation).

	DM		ND	
	*n*	% of *n*	*n*	% of *n*
Total sample (*n*)	22	—	47	—
Sex				
Female	9	41%	29^a^	62%
Male	13	59%	18^a^	38%
Age				
Average	71.9 ± 9.3	—	73 ± 7.7	—
Range	46–83	—	50–88	—
Height [cm]	169 ± 11	—	171 ± 9	—
Weight [kg]	91 ± 19	—	78 ± 18	—
BMI	31.8	—	26.7	—
Origin				
Australian	3	14%	18	38%
European/Caucasian	4	18%	13	28%
East‐Asian	2	9%	1	2%
Other	0	0%	1	2%
Not specified	13	59%	14	30%
Neuropathy confirmed	11	50%	11	23%
Actively moving hours/day				
0–2	5	23%	8	17%
3–4	5	23%	11	23%
5–6	8	36%	15	32%
7–8	4	18%	7	15%
9+	0	0%	6	13%
Difficulty finding comfortable shoes	14	64%	24	51%
Difficulty finding good‐fitting shoes	13	59%	21	45%

^a^ indicates *p* < 0.05.

### 3.2 Statistical Shape Models (SSMs)

**FIGURE 2:**
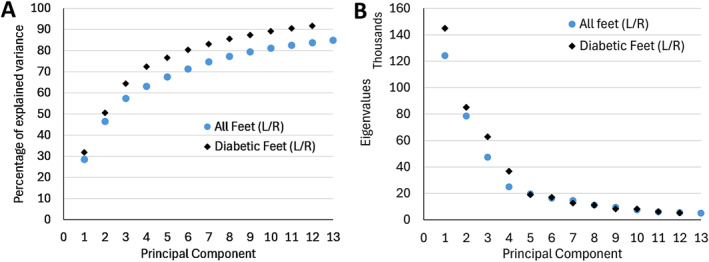
(A) Percentage of explained variance by the first 13 principal components for both SSM groups. (B) Corresponding eigenvalues for both SSM groups.

When including all feet in the SSM, the first 13 PCs accounted for 83.8% of the variability with each PC contributing to at least 1% of the variance (Figure 2A). For the SSM consisting only of foot scans from people with DM, the first 12 PCs accounted for 91.7% of the variance and at least 1% of the variance. The first 3 PCs accounted for more than half of the variability in the combined (all feet) group (57.4%) and in the DM group (64.3%). The cumulative eigenvalues for the two groups show similar magnitudes of variation in relation to the PCs, with the diabetic foot showing slightly more variability in the first PCs than all the feet analyzed together (Figure 2B).

**FIGURE 3:**
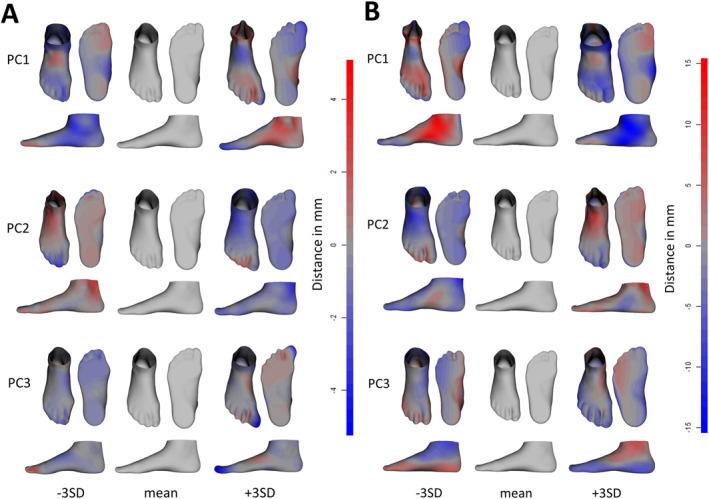
Foot shapes for analyses with (A) nondiabetic feet and (B) diabetic feet for the first 3 PCs across ± 3 standard deviations. Blue indicates where the PC foot shape is larger than the mean foot and red indicates where the PC foot shape is smaller than the mean foot.

In the ND and DM populations, PC1 accounted for variability in ankle width, arch height, and in respect to an edematous foot (−3SD for ND and +3SD for DM) or an atrophic foot with reduced soft tissue prominence (+3SD for ND and −3SD for DM) (Figure 3). PC1 for the ND population also showed more variation in the hallux (smaller and adducted for −3SD and larger and abducted for +3SD). PC2 showed the shift from an adducted forefoot and higher arch (+3SD for ND and −3SD for DM) to an abducted forefoot and flatter arch (−3SD for ND and +3SD for DM). For the ND population, PC2 accounted for the foot type from Egyptian to Roman to Greek (− to + SD). PC3 accounted for an abducted and larger hallux, wider toe splay, and a larger forefoot width in relation to the heel width in the +3SD range. For the ND population, PC3 also showed a larger hallux toe height (+3SD). In all three PCs, the variations from the mean were greater for DM feet. Overall, the mean diabetic foot showed a lower arch and more abducted hallux than the mean ND foot.

**FIGURE 4:**
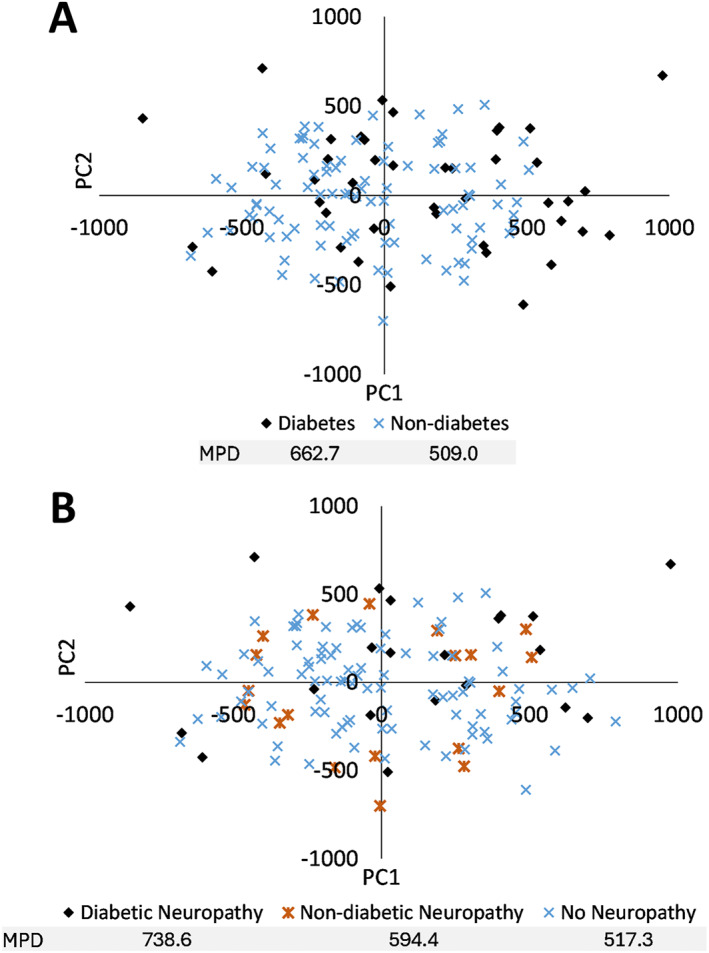
PC1 and PC2 scores distinguishing between feet from (A) people with diabetes and no diabetes and (B) people with diabetic neuropathy, nondiabetes related neuropathy, and no neuropathy. The mean pairwise distance (MPD) is shown under each population.

**FIGURE 5:**
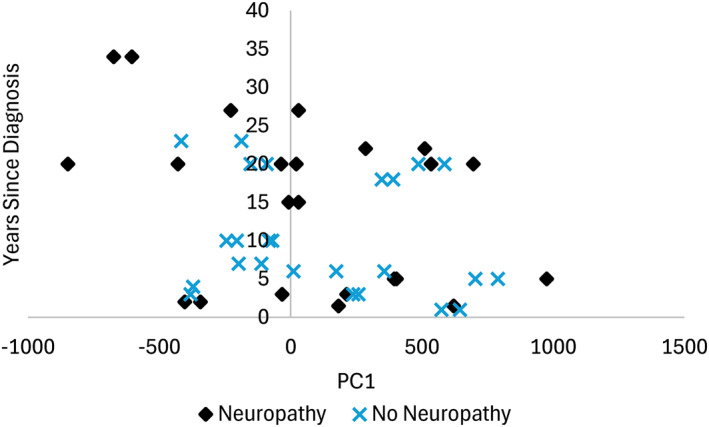
The years since diagnosis for people with diabetes with respect to PC1.

Visual cluster analysis of PC1 and PC2 of the SSM with all feet combined showed that the DM feet accounted for most of the values on the perimeter of the cluster whereas some DM feet fell within the middle of the cluster (Figure 4A). The mean pairwise distance (MPD) between the points within the DM cohort was 30% larger than the MPD of the ND cohort. When further distinguishing between diabetic neuropathy, nondiabetic neuropathy, and no neuropathy, the cluster showed that those with diabetic neuropathy have the largest spread across PC1 and PC2, as shown by a 43% larger MPD than for people without neuropathy, regardless of diabetes diagnosis, and 24% larger MPD than for people without diabetes but with neuropathy (Figure 4B). People without diabetes and neuropathy had an MPD 15% larger than those without neuropathy (regardless of diabetes diagnosis). A general downward trend was observed when considering years since diagnosis with PC1 for people with diabetes and neuropathy (*R* ^2^ = 0.11), but no clear trend was seen for people with diabetes and neuropathy (*R* ^2^ = 0.07) (Figure 5).

### 3.3 Partial Least Squares Regression (PLSR)

**FIGURE 6:**
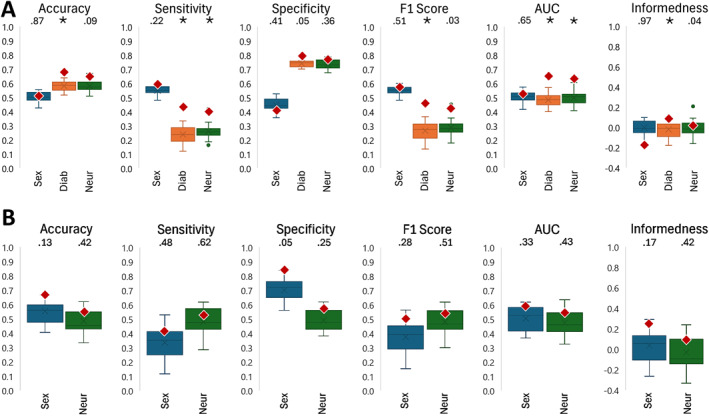
The classification of the prediction model (in red diamonds) compared to the random model (in box plots) for the model accuracy, sensitivity, specificity, F1 score, area under the receiver operating characteristic curve (AUC), and the bookmaker informedness for (A) all feet together with sex, diabetes, and neuropathy characteristics and (B) only the diabetic foot model with sex and neuropathy characteristics. *p*‐values for the comparison of the classification model result with the random label results are given above each plot, * indicates *p* < 0.01. Diab = presence of diabetes; Neur = presence of neuropathy.

The PLSR for the total population comprised 132 feet and the diabetic PLSR 42 feet. When including all 132 feet, 27 components were necessary to explain at least 90% of the variability [21]. After the LOO validation, the PLSR, including all feet, showed statistically significant accuracy, sensitivity, F1 score, AUC, and bookmaker informedness for predicting presence of diabetes and sensitivity and AUC for the presence of neuropathy relative to the 20 randomly generated models (Figure 6A). For the model with only diabetic feet, there were no statistical differences shown for the ability to predict sex and neuropathy after the LOO validation (Figure 6B). *R* ^2^ values for the continuous variables were calculated before and after the LOO validation (Table 2).

**TABLE 2:** *R* ^2^ values for PLSR before and after the leave‐one‐out (LOO) validation.

	Before LOO		After LOO	
	All	Diabetic	All	Diabetic
Age	0.92	0.92	0.15	−0.05
BMI	0.86	0.91	−0.06	−0.3
Years since diagnosis	0.9	0.91	−0.011	0.15

## 4 Discussion

Qualitative and quantifiable differences between the foot shape of older adults with and without DM and with and without neuropathy were determined based on anatomical and personal characteristics. The SSM showed larger variability in foot shape among individuals with diabetes and neuropathy than those without one or two of these diagnoses. The PLSR results showed evidence that the presence of diabetes may be determined based on foot shape features and weakly by age, BMI, and years since diabetes diagnosis. Given that people with DM generally have foot shapes with more extreme features farther from the “average” foot shape, this research shows that it may be more appropriate to create multiple shoe lasts for people with DM to account for multiple deformity dimensions. These lasts may be characterized by the extreme shapes in both directions (− and +SD), though future research may further specify last designs.

The PCs of the SSMs accounted for the main foot shape differences across the populations. For both ND and DM populations, these differences included changes in ankle width (PC1), arch height and associated instep height (PC1/PC2), edematous/atrophic foot (PC1), ab/adducted forefoot (PC2) or hallux (PC3), hallux size (PC3), toe splay (PC3), and forefoot width in relation to heel width (PC3). These three PCs accounted for 57% and 63% of the variation in the ND and DM populations, respectively, which is similar to the variation accounted for in the first three PCs in previous research with healthy feet [24]. The order of the shape features' significance is also similar to previous research, which showed arch height as a primary foot morphology difference, followed by foot width, hallux orientation, foot type, and midfoot width [17]. The variations in instep height and width (forefoot and heel) are consistent with previous research that showed differences according to person origin, which could have been a contributing factor in this work [26]. Furthermore, the similarities in shape differences compared to previous studies on healthy feet [17, 19, 27] show that the sampled feet are representative of the population. Moreover, the age difference between the current sample (46–88 years old) and previous samples (e.g., 18–40 years old in ref. [19]) and the presence of pathology as opposed to healthy feet show that variations in foot shape remain relatively consistent with increasing age and the onset of pathology such as type 2 diabetes. Similarly, Stanković and colleagues [18] observed that feet with the same clinical classification of common foot deformities nevertheless exhibited a high shape variability.

The SSMs revealed differences between the DM and ND populations. For the ND population only, PC1 showed variations in hallux size and ab/adduction and PC3 revealed changes in hallux height. The plots of the PC scores for individuals with non‐diabetic neuropathy formed an ellipse between those with diabetic neuropathy and those without neuropathy, showing that neuropathy may affect foot shape even when diabetes is not necessarily the cause. These PCA results show that, even though there may not be distinct foot shape differences between individuals with diabetes and neuropathy, these populations represent the more extreme cases of variations in foot shape. Furthermore, as the years since diagnosis increase, there are more extreme PC scores as shown in PC1.

The PLSR was able to predict certain latent variables associated with the populations. After the LOO validation in the model with all the feet, the accuracy, sensitivity, F1 score, AUC, and bookmaker informedness showed statistically better predictions for the presence of diabetes than the random model. Similar results were found for sensitivity and AUC for the presence of neuropathy in the DM population. However, considering that the AUC for both predicting diabetes and neuropathy were below 0.7, the sample size should be increased. For the DM population, the model showed that there could be a trend toward predicting the sex of the individual better than a random model, which is consistent with previous research showing differences in shape between male and female feet [17, 26].

The PLSR with only the DM population required fewer components to reach the variability threshold than when including all feet, but the results were less indicative of an ability to determine sex and neuropathy than the random models. As the PCA revealed that there were differences in the clustering of diabetic neuropathy versus nondiabetic neuropathy, the relationship between foot shape changes and neuropathy in general may be more complicated and require a larger sample size for exploration. In the PLSR models, prior to applying the LOO validation, age, BMI, and years since diagnosis exhibited strong correlations with foot shape prediction. However, following the LOO validation, these variables proved to be noninformative, suggesting that PLSR overfitting may lead to misleading results if not properly mitigated.

A few limitations worth noting: Equal population sizes from individuals with and without diabetes would have strengthened the results. The tests for this study were conducted in one geographical location and consisted mainly of individuals of European descent. As there was no option on the questionnaire to classify between Australian by European descent or native Aboriginal descent, future studies may increase the sample size and specify origin with more detail. It may also be beneficial to include data from different regions globally, which could consider different anthropometric origins [26]. Furthermore, all questionnaires were completed by the patients and thus limited medical accuracy; future studies may include detailed medical records providing exact neuropathy scores and indicating other factors influencing foot shape features.

This research seeks to provide shape change information that may be a starting point for future work to create predictive 3D diabetic foot shape models; these models may eventually be used for clinical interpretation, but the current research gatherings should be used with caution. Future models could also explore trait‐specific variations, such as ab/adduction of the hallux and forefoot versus heel width, to create various characteristic‐specific shoe lasts. Given that people with neuropathy even without diabetes showed more extreme shapes than those without neuropathy, there is a potential for neuropathy‐specific shoe lasts in addition to diabetic‐specific designs. Furthermore, future work may implement PLSR and PCA to create more robust models of the feet of various populations to better understand foot shape changes in relation to personal characteristics.

## 5 Conclusion

Overall, this study showed that PCA and PLSR are valuable for demonstrating foot shape differences in people with and without diabetes and neuropathy. These results may inform shoe last design for people with diabetes and/or neuropathy to prevent ulcer formation or growth. Future work may expand the analysis and sample size to create more comprehensive foot shape models specific to pathology, and from these expanded foot shape databases, more accurate shoe last designs may be created for different populations.

## Author Contributions

**Sarah L. Hemler:** conceptualization, data curation, formal analysis, funding acquisition, methodology, project administration, visualization, writing – original draft, review and editing. **Robert W. Schuster:** data curation, formal analysis, methodology, software, validation, writing – review and editing. **A.‐V. Behling:** methodology, software, writing – review and editing. **Z. Pataky:** conceptualization, supervision, writing – review and editing. **L. A. Kelly:** conceptualization, formal analysis, funding acquisition, methodology, project administration, resources, supervision, visualization, writing – review and editing.

## Conflicts of Interest

The authors declare no conflicts of interest.

## Data Availability

The datasets generated and/or analyzed during the current study are available upon request.
